# Application of Mutual Information-Sample Entropy Based MED-ICEEMDAN De-Noising Scheme for Weak Fault Diagnosis of Hoist Bearing

**DOI:** 10.3390/e20090667

**Published:** 2018-09-04

**Authors:** Fen Yang, Ziming Kou, Juan Wu, Tengyu Li

**Affiliations:** 1 School of Mechanical Engineering, Taiyuan University of Technology, Taiyuan 030024, China; 2 Shanxi Province Mineral Fluid Controlling Engineering Laboratory, Taiyuan 030024, China; 3 National-Local Joint Engineering Laboratory of Mining Fluid Control, Taiyuan 030024, China

**Keywords:** weak fault, hoist bearing, improved complete ensemble empirical mode decomposition, minimum entropy deconvolution, sample entropy, mutual information

## Abstract

In this paper, a novel weak fault features extraction scheme is proposed to extract weak fault features in head sheave bearings of floor-type multi-rope friction mine hoists in strong noise environments. A mutual information-based sample entropy (MI-SE) is proposed to select the effective intrinsic mode function (IMF). The numerical simulation presented in this paper has demonstrated that the improved complete ensemble empirical mode decomposition with adaptive noise (ICEEMDAN) has a poor performance on weak signals processing under a strong noise background, and fault features cannot be identified clearly. The de-noised signal is decomposed into several IMFs by the ICEEMDAN method, with the help of the minimum entropy deconvolution (MED), which works as a pre-filter to increase the kurtosis value by about 3.2 times. The envelope spectrum of the effective IMF selected by the MI-SE method shows almost all fault features clearly. An analogous experiment system was built to verify the feasibility of the proposed scheme, whose results have also shown that the proposed hybrid scheme has better performance compared with ICEEMDAN or MED on the weak fault features extraction under a strong noise background. This paper provides a novel method to diagnose the weak faults of the slow speed and heavy load rolling bearings in a strong noise environment.

## 1. Introduction

The head sheave is a critical part of the floor-type multi-rope friction hoist system in coal mines, and its health status has an important influence on the entire performance of the hoisting system. Once a fault occurs, it will cause huge risks and economic losses to the coal mine. Therefore, it is of great significance to carry out research on the fault diagnosis of head sheave bearings. Due to the fact that the bearings work at a slow speed (from 40 rpm to 60 rpm) with heavy loads (e.g., tens or hundreds of kN magnitude), the fault vibration signal is relatively weak. This is especially true for the incipient fault, as it is easily submerged by environment noise. Though numerous technologies have been proposed to diagnose faults [1,2,3], how to extract the weak useful fault features is still facing challenges. In order to ensure the safety of the hoist system, it is necessary to use appropriate noise reduction techniques to extract the weak fault features of the bearing accurately from vibration signals in a strong noise environment.

The typical noise reduction methods for fault diagnosis include traditional filtering, wavelet de-noising, and empirical mode decomposition (EMD), and so on. The wavelet de-noising technology has the advantage of multi-resolution and has been used in the weak fault features extraction of rolling bearings [4,5]. However, as a non-adaptive signal processing method, the selections of basic functions and thresholds are determined by experience, which may weaken the performance of the wavelet de-noising technology. EMD is a nonlinear adaptive signal processing method proposed by Huang [6], which can decompose a complex signal into finite transient intrinsic mode functions (IMF). Since it has a good adaptability, it has been widely applied in the field of fault diagnosis [7,8,9]. However, this method has drawbacks such as mode mixing and spurious modes during the decomposition process, due to its work principle. Wu et al. [10] proposed the ensemble empirical mode decomposition (EEMD) method, which performs decomposition over an ensemble of noisy copies of the original signal and obtains the final results by averaging the corresponding IMFs. However, the problem of mode mixing can be relieved to a certain extent by adding white Gaussian noise [11,12,13,14], and the artificially added white noise cannot be completely removed; the number of IMFs produced by different realizations of signal plus noise is often inconsistent resulting from the algorithm. The complementary ensemble empirical mode decomposition (CEEMD) method [15] significantly alleviates the signal reconstruction problem by adding pairs of complementary noises (i.e., adding and subtracting) to the original signal, but it cannot guarantee that the two pairs of signals plus noise can produce the same number of IMFs. The complete ensemble empirical mode decomposition with adaptive noise (CEEMDAN), which was proposed by Torres [16], can recover the completeness property of EMD, and has proven to be a significant improvement on EEMD. It effectively overcomes the problems that exist in EEMD and CEEMD, and is widely applied in areas such as biomedical engineering [17], seismic fields [18], and so on. In order to further remove the residual noise in the IMFs and the spurious IMFs during the decomposition process, Colominas [19] introduced the improved complete ensemble empirical mode decomposition with adaptive noise (ICEEMDAN) method, which solved the problem of mode mixing and spurious modes, and obtained good results in motor failure analysis [20]. However, under strong noise background, the ICEEMDAN method has poor performance for the signal, mixed with a lot of noise. The weak fault features cannot be extracted effectively, due to the interference of strong noise. The minimum entropy deconvolution (MED) has a strong noise reduction ability. It can enhance a few large spikes in the signal, to obtain a signal with the maximum kurtosis, which will decrease the influence of the noise on the ICEEMDAN effectively.

In the signal processing of the ICEEMDAN method, it is important to select the effective IMFs from a series of IMFs generated by ICEEMDAN. Mutual information (MI) and sample entropy (SE) are often used as the criterion for selecting the effective IMFs [21,22]. The MI represents the relevance of two variables; the more relevant they are to each other, the greater the MI that will be achieved. SE can be used to measure the complexity of a time series; the more complex the signal is, the greater SE will be obtained. In a strong noise environment, the original fault signal often contains a lot of noise, which leads to the problem that there is too much noise in the high-frequency IMFs, and that a number of spurious IMFs may exist in the low or middle frequency in the decomposition of ICEEMDAN. If the effective IMFs are only selected based on the MI between every IMF and the original signal, the noisy IMFs in high frequency will be treated as effective IMFs, resulting in the fault features being unable to be extracted successfully. Similarly, if the effective IMFs are selected only based on the SE of IMFs, spurious IMFs may be treated as effective IMFs.

In order to select the more reliable and effective IMFs in a strong noise environment, the mutual information-based sample entropy (MI-SE) method is proposed to overcome the shortcomings of MI or SE. For the poor performance of ICEEMDAN used to extract the weak fault features of the bearing in strong noise environment, this paper uses the MED method to de-noise the original signal firstly, then the pretreated signal is decomposed by ICEEMDAN into a series of IMFs. Finally, the effective IMFs are selected based on the proposed MI-SE method, and the fault features are obtained successfully.

## 2. Basic Theory

### 2.1. Minimum Entropy Deconvolution

**Figure 1:**
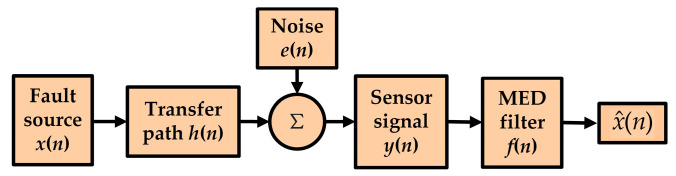
Noise reduction of bearing based on minimum entropy deconvolution (MED).

As an adaptive noise reduction method, the MED, proposed by Wiggins [23], will introduce an inverse filter *f*(*n*) to magnify a few large spikes in the original signal to obtain a signal with the maximum kurtosis, as illustrated in Figure 1. This characteristic can highlight the shock well, so, the MED method is very suitable for the noise reduction of the rolling bearings.

During the transmission of the shock signal *x*(*n*) generated by the defective rolling bearing through the transfer function *h*(*n*), various types of noise *e*(*n*) may mix in and attenuate *x*(*n*) to *y*(*n*). Obviously, the original features of signal *x*(*n*) are submerged by strong noise, with its entropy becoming larger. From Figure 1, the vibration signal can be expressed as:

(1) y(n)=h(n)∗x(n)+e(n) 

By using the MED method, one has:

(2) $$\hat{x}(n)=f(n)*y(n)\;$$

where $\hat{x}(n)$ is an estimate of *x*(*n*); the closer they are, the better the inverse filter *f*(*n*) is, and a smaller entropy of $\hat{x}(n)$ will be obtained. The normalized fourth-order cumulate of $\hat{x}(n)$ was used by Wiggins to measure its size, which can be expressed as:

(3) $$O^{4}(f(n))=\frac{\sum_{i=1\;}^{N}{\hat{x}}^{4}(i)}{{\left[\sum_{i=1}^{N}{\hat{x}}^{2}(i)\right]}^{2}}$$

The inverse filter *f*(*n*) is optimal when the normalized fourth-order cumulate value of $\hat{x}(n)$ is the maximum, which is:

(4) $$\frac{\partial O^{4}(f(n))\;}{\partial f(n)}=0$$

The optimal inverse filter *f*(*n*) can be obtained from the Equations (1)–(4), and the signal with the maximum kurtosis will be realized.

### 2.2. Improved Complete Ensemble EMD

**Figure 2:**
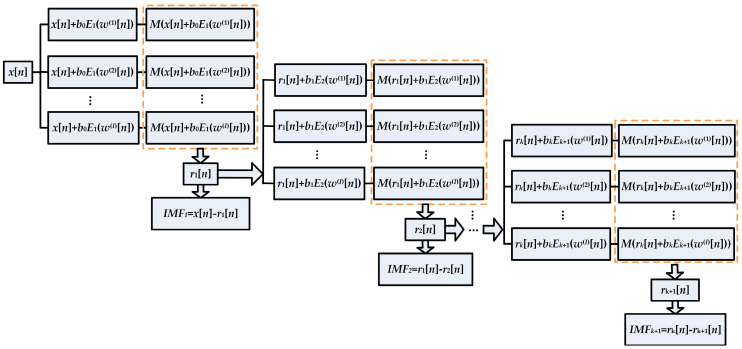
Flow chart of the improved complete ensemble empirical mode decomposition (ICEEMDAN).

The ICEEMDAN method has great improvement on the ensemble EMD and complete ensemble EMD methods. For simplicity, $E_{k}(\cdot )$ is defined as the operator for producing the *k*th IMF that is generated by EMD, and M(⋅) is the operator for calculating the local mean; for example, *E*_1_(*x*) = *x* − *M*(*x*), and *w*^(*i*)^ (*I* = 1, 2, …, *I*) is a realization of unit variance white Gaussian noise with a zero mean. The flow chart of the ICEEMDAN algorithm can be described and shown in Figure 2 [19].

### 2.3. Mutual Information Based Sample Entropy

Usually, a series of IMFs will be generated after ICEEMDAN decomposition. In the strong noise environment, there will be many spurious IMFs and noisy IMFs due to the noise interference. Mutual information (MI) and sample entropy (SE) can be used to select the effective IMFs. The MI is used to indicate the correlation between two random variables [21], and is actually a special case of the relative entropy. The MI of two variables *X* and *Y* is defined as:

(5) $$I(X,Y)=\iint p(x,y)\cdot \log\frac{p(x,y)\;}{p(x)\cdot p(y)}dxdy$$

where *p*(*x*) and *p*(*y*) are the probabilistic density functions of *X* and *Y*, respectively, and *p*(*x*,*y*) is their joint probabilistic density function.

SE is the complexity metric of a time series, whose value will be larger if the signal is more complex. The SE of a signal can be determined by [22]:

(6) $$SE(m,r,N)=\ln[\frac{1}{N-m+1\;}\sum_{i=1}^{N-m+1}C_{r}^{m}(i)-\frac{1}{N-(m+1)+1}\sum_{i=1}^{N-(m+1)+1}C_{r}^{m+1}(i)]$$

where *N* is the signal length, *m* is the match of length, *C_r_^m^*(*i*) represents the probability that two epochs match each other, and *r* is the noise filter parameter, which is usually chosen to be around 25% of the standard deviation of the signal.

## 3. Weak Fault Features Extraction Based on MED-ICEEMDAN

**Figure 3:**
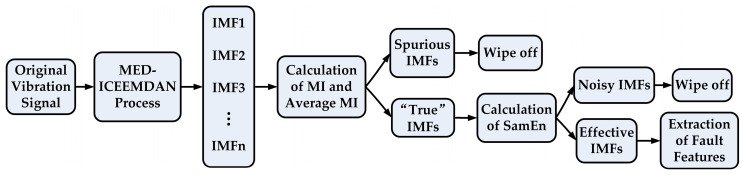
Flow chart of the proposed weak fault features extraction method.

In order to extract weak fault features in the strong noise environment, a hybrid scheme based on MED-ICEEMDAN is proposed here to reduce the noise and to increase the signal-to-noise ratio (SNR). MED can enhance a few large spikes in the signal and make it have the maximum kurtosis, so that the SNR of the signal is improved despite its poor performance in protruding weak signals that are submerged by noise. The ICEEMDAN can effectively eliminate the extra artificially added white noise in the IMFs, and it has better performance than EMD, EEMD, and CEEMDAN in overcoming mode mixing. However, for the signal mixed with a lot of strong environment noise, the ICEEMDAN method is unable to extract fault features effectively, which will be illustrated in Section 4. In order to reduce the effect of noise on the ICEEMDAN method, the original signal is pretreated with the MED method, and then the ICEEMDAN method is used to decompose the pretreated signal into numerous IMFs. The “true” IMFs, representing a simple oscillation mode with a physical meaning, can be selected based on the fact that the MI between the spurious IMFs and the original signal is much smaller than that between the “true” IMFs and the original signal, which will be specified in Section 4. Usually, the average MI is defined as the criterion to determine the “true” IMFs, (which are larger than the average MI). However, if the original signal contains strong noise, the “true” IMFs selected based on MI may contain much noise, which will affect the extraction of the fault features. In order to ameliorate the dissatisfactory situation, the SE values of the “true” IMFs selected based on MI are calculated to remove the noisy IMFs according to the larger SE, which means a more complex or more irregular sequence, such as a noise signal. In this paper, the IMFs selected based on the MI-SE method are the final effective IMFs, from which the weak fault features can be derived. Using the MI-SE method as the criterion for selecting effective IMFs, can overcome the blindness of the traditional ICEEMDAN selecting the first IMF directly as the effective IMF after the decomposition, which makes the obtained result more reliable. By taking advantages of these methods, the weak fault features of the bearings in a strong noise environment can be extracted successfully. The specific flow chart is illustrated in Figure 3.

## 4. Simulation Analysis of Faulted Rolling Bearings Based on MED-ICEEMDAN

Once a failure has occurred, the defect will cause periodical impact vibration when the balls pass through the defect on the inner or outer race with low frequency, which is called the “pass frequency”. The theoretical passing frequency of the corresponding fault can be expressed as Equation (7):

(7) $$\left\{ \begin{array}{l} f_{o}=\frac{N_{b}\;}{2}\left(1-\frac{B_{d}}{P_{d}}\cos\theta \right)f_{r}\\ f_{i}=\frac{N_{b}}{2}\left(1+\frac{B_{d}}{P_{d}}\cos\theta \right)f_{r}\\ f_{b}=\frac{P_{d}}{2B_{d}}\left(1-\frac{B_{d}^{2}}{P_{d}^{2}}\cos^{2}\theta \right)f_{r} \end{array}\right.$$

where *f_o_* and *f_i_* are the ball pass frequencies of the outer race and the inner race, respectively. *f_b_* is the ball spin frequency, *f_r_* is the rotational frequency, *N_b_* is the number of balls, *B_d_* is the diameter of the ball, *P_d_* is the diameter of the bearing pitch circle, and *θ* is the bearing contact angle.

Take the inner race defect as an example for theoretical analysis. Since the relative position between the defect point and the test point changes with the rotation of the inner race, the periodical impact force generated by the defect is modulated by the rotation of shaft, that is:

(8) $$f(t)=\sum_{n=0\;}^{N-1}D\cos(2\pi f_{r}t)\delta (t-nT_{0})$$

Then the vibration signal of the rolling bearing can be expressed as:

(9) $$x(t)=f(t)*h(t)=\sum_{n=0\;}^{N-1}\left[D\cos(2\pi f_{r}t)\delta (t-nT_{0})\right]*\left[Ae^{-\xi 2\pi f_{n}t}\cos(2\pi f_{d}t-\theta )\right]$$

where *h*(*t*) = *Ae* ^−*ξ*2*πfnt*^cos(2*πf_d_t* − *θ*) represents the impulse response of the rolling bearing under unit impulse force, *ξ* is the damping ratio of the system, and *f_n_* and *f_d_* are the natural frequency and damped natural frequency of the system, respectively. 

**Figure 4:**
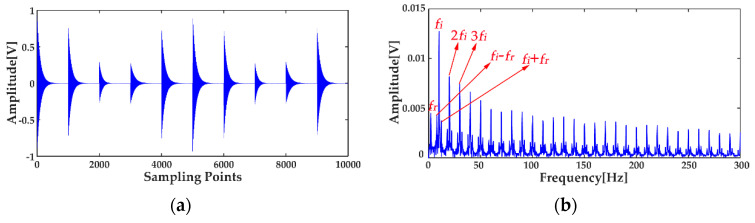
Simulation signal of bearing inner race fault without noise: (**a**) time waveform; (**b**) envelope frequency spectrum.

**Figure 5:**
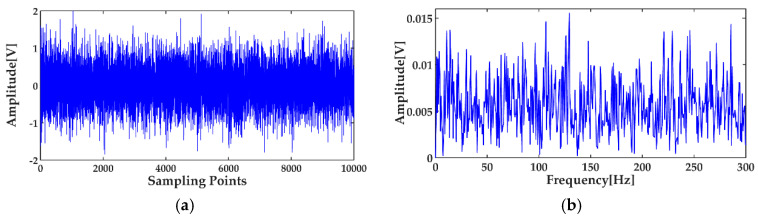
Simulation signal of bearing inner race fault with noise: (**a**) time waveform; (**b**) envelope frequency spectrum.

**Figure 6:**
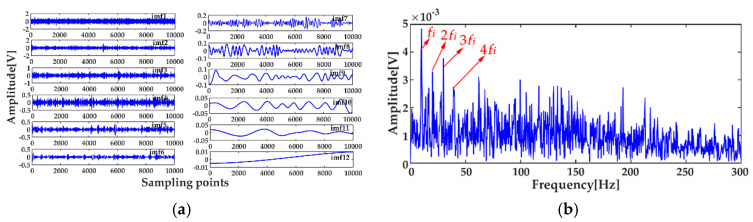
Decomposition results using ICEEMDAN: (**a**) decomposition results; (**b**) envelope frequency spectrum of the effective intrinsic mode function (IMF).

**Figure 7:**
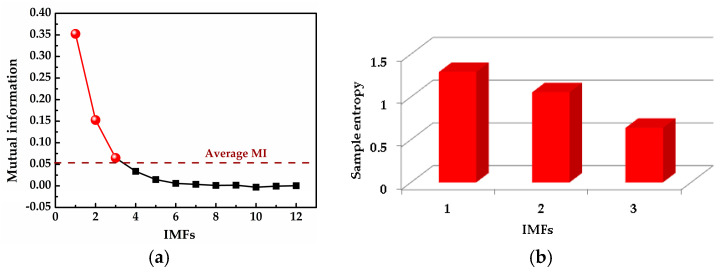
Calculation results of mutual information (MI) and sample entropy (SE): (**a**) calculation results of MI for all IMFs; (**b**) calculation results of SE for the “true” IMFs.

In the theoretical simulation, a pulse signal with a time interval *T_i_* of 0.1 s (*f_i_* = 10 Hz) was used to simulate the periodical impact vibration generated by the bearing inner race defect. Assuming that the natural frequency of the bearing system is *f_n_* = 1000 Hz, then the simulation signal can be expressed as (10) according to Equation (9):

(10) $$\left\{ \begin{array}{l} x_{1}(t)=\cos(2\pi t)\times e^{-100t\;}\sin(2000\pi t)\\ x_{2}(t)=x_{1}(t)+n(t) \end{array}\right.$$

where *x*_1_(*t*) and *x*_2_(*t*) are the simulation vibration signals of the bearing inner race fault without and with noise, respectively, and *n*(*t*) denotes the Gaussian white noise. The time waveforms are shown in Figure 4a and Figure 5a, and the corresponding envelope frequency spectrums are shown in Figure 4b and Figure 5b. When there was no noise in the signal, the fault frequency, multiple frequencies, and side frequencies of the bearing inner race were all clear, as shown in Figure 4b, and their amplitude keeping decreased in general. However, when the signal was mixed with strong noise, neither the time waveform nor the envelope frequency spectrum of the signal showed any useful information about the bearing inner race fault. By using ICEEMDAN, the signal in Figure 5a was decomposed and it is shown in Figure 6a. The MI values between every IMF and the original signal were calculated, as shown in Figure 7a. The dashed line represents the average MI, which was 0.0523. So, the first three IMFs were the “true” IMFs according to the characteristics of the MI. The SE values of the first three IMFs are shown in Figure 7b. Based on the proposed MI-SE method, IMF3 was selected as the effective IMF, and its envelope spectrum is shown in Figure 6b. Compared with Figure 5b, it can be seen that although the bearing inner race fault features could be identified, it was still not ideal to directly use the ICEEMDAN when the signal was mixed with a lot of noise.

**Figure 8:**
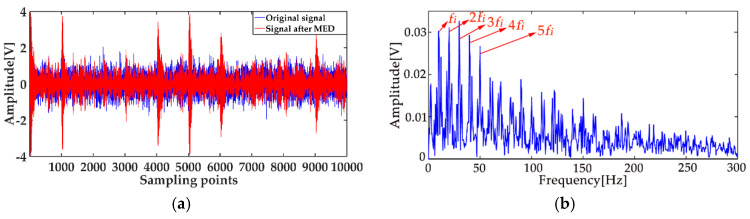
De-noised signal using MED: (**a**) comparison between the de-noised signal by MED and the original signal; (**b**) envelope spectrum of signal after MED noise reduction.

**Figure 9:**
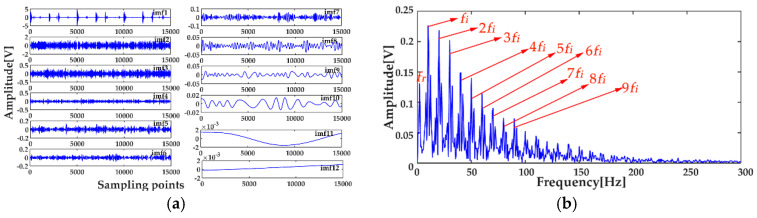
Decomposition results using MED-ICEEMDAN: (**a**) decomposition results; (**b**) the envelope frequency spectrum of the effective IMF.

**Figure 10:**
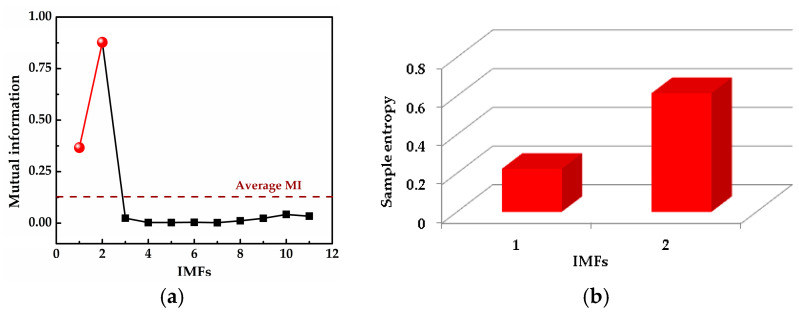
Calculation results of MI and SE: (**a**) calculation results of MI for all IMFs; (**b**) calculation results of SE for the “true” IMFs.

In order to reduce the influence of the noise on the weak fault features extraction by using ICEEMDAN, the MED method was proposed as a prefilter to de-noise the original signal. Figure 8a shows the comparison results between the de-noised signal by MED, and the original signal. It can be seen that the shocks in the noisy signal became prominent after the noise reduction by MED, and the kurtosis value of the signal increased from 3.09 to 9.79. However, some weak impacts were still submerged by the noise, which was the limitation of the MED method. The envelope spectrum of the signal after MED noise reduction is shown in Figure 8b, from which one can see that some multiple frequencies and side frequencies were not protruded. Then, by using ICEEMDAN, the pretreated signal can be decomposed, and the result is shown in Figure 9a. The MI values between every IMF and the original signal were calculated, and they are shown in Figure 10a, from which one can see that the average MI was 0.1264. According to the characteristics of MI, the first two IMFs were the “true” IMFs. Figure 10b shows the SE values of the first two IMFs. Based on the proposed MI-SE method, IMF1 was selected as the effective IMF, and its envelope spectrum is shown in Figure 9b. Compared with the envelope frequency spectrums obtained by aforementioned methods, the results obtained by using MED-ICEEMDAN better conformed to the case without noise, and most of the fault features were obtained, which proves that the method proposed in the paper is effective. In order to further verify the actual reliability of this method, an experimental study was carried out.

## 5. Experiments and Results

**Figure 11:**
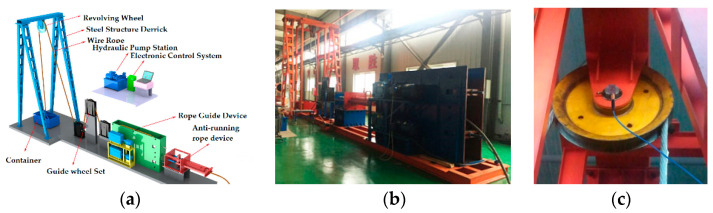
Experimental equipment: (**a**) components of the equipment; (**b**) photo of the equipment; (**c**) locations of acceleration sensors.

**Figure 12:**
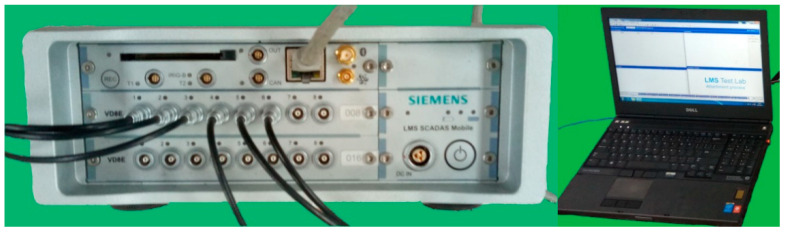
Data acquisition equipment.

As the coal mine hoist is a large-scale rotating equipment, there are strict requirements for the security assurance, so it is difficult to carry out the field experiment on the mine hoist head sheave bearings. Therefore, an analogous experiment was carried out to verify the reliability of the proposed MED-ICEEMDAN method in the extracting weak fault features of these bearings in a strong noise environment. In order to simulate the operating conditions of the head sheave bearings more realistically, a hoist testing setup, as shown in Figure 11, was built based on its working principles and load characteristics. The testing system mainly consists of a wire rope, a conveyance, a vertical guide frame, a guide wheel, a revolving wheel device (including rolling bearings), driving mechanisms, a rope guide device and a hydraulic pump station. According to the experimental requirements, a steel structure derrick with a height of 10 m was built on the ground to simulate the derrick of the actual hoist, and the revolving wheel device was used to simulate the hoist head sheaves. There was no balance rope, due to the limited lifting distance of the simulation test bench. Therefore, this experiment setup converted the near-vertical motion of the wire rope, which should be placed on the friction wheel in a practical floor-type multi-rope friction mine hoist, into a horizontal motion, through a set of guide wheels and a rope traction drive device, to control the movement of the conveyance. Through this testing system, the vibration signals were tested under three states: the normal condition, the inner race defect, and the outer race defect of the bearing. A single point with a diameter of 0.5 mm was machined on the inner and outer races of the test bearing respectively, using the electro-discharge technology to simulate the incipient spalling failure of the bearing. The vibration data of the bearing was acquired by a three-way accelerometer with a frequency response range of 1–5 kHz (PCB356A26, PCB Piezotronics, lnc. Buffalo, New York, NY, USA). The sensor was installed on the housing of the bearing cover, as shown in Figure 11c. The signal acquisition device was a multi-functional 16-channel data acquisition system (LMS SCADAS Mobile, LMS, Leuven, Belgium), and this system was driven and controlled by the signal acquisition software (LMS Test. Lab, LMS, Leuven, Belgium), as shown in Figure 12.

The bearings used in the experiment were deep groove ball bearings, and their parameters are shown in Table 1.

The rotating frequency *f_r_* was about 3.2 Hz, calculated based on the wire rope running speed (which is 3 m/s) and the radius of the revolving wheel (which is 150 mm). Through Equation (7), the corresponding fault frequencies of the bearing inner and outer races were *f_i_* = 15.83 Hz and *f_o_* = 9.7696 Hz, respectively.

**Figure 13:**
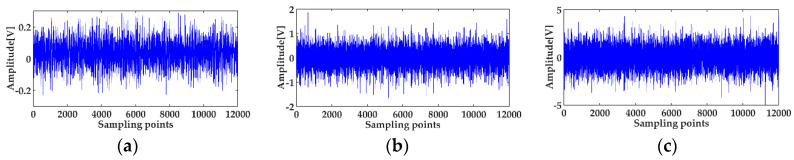
Vibration signal of the three states: (**a**) normal status; (**b**) the fault signal of the bearing inner race; (**c**) the fault signal of the bearing outer race.

**Figure 14:**
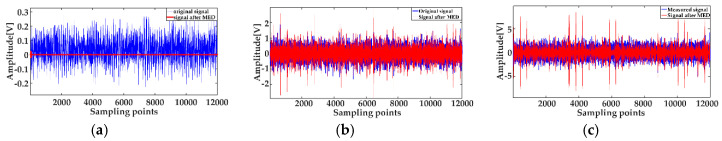
Comparison of the signal de-noised by MED in the red line, and the original signal in the blue line of three statuses: (**a**) normal status; (**b**) the fault signal of the bearing inner race; (**c**) the fault signal of the bearing outer race.

**Figure 15:**
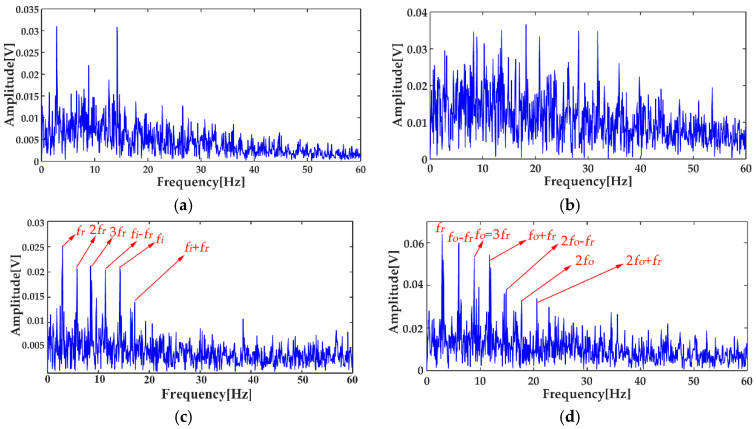
Envelope spectra of fault signals: (**a**) the bearing inner race fault signal before the MED noise reduction; (**b**) the bearing outer race fault signal before the MED noise reduction; (**c**) the bearing inner race fault signal after the MED noise reduction; (**d**) the bearing outer race fault signal after the MED noise reduction.

The vibration signals of the three states are shown in Figure 13, which were digitized at a sampling frequency of 12 kHz. As can be seen from Figure 13, it was not possible to judge whether the bearing was faulty, and where the location of the defect was, by the time waveforms with noise interference. The signals for three statuses after de-noising using the MED method, are shown in Figure 14. It can be seen that the amplitude of the normal bearing signal after MED noise reduction was very small, while the shocks of the inner and outer races fault vibration signals became prominent, but some weak shocks were still submerged by noise. The envelope spectra of the fault status before and after the MED noise reduction are shown in Figure 15.

**Figure 16:**
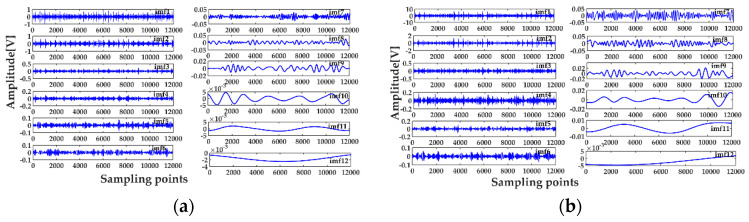
Decomposition results of the bearing fault signals by MED-ICEEMDAN: (**a**) the result of the inner race; (**b**) the result of the outer race.

**Figure 17:**
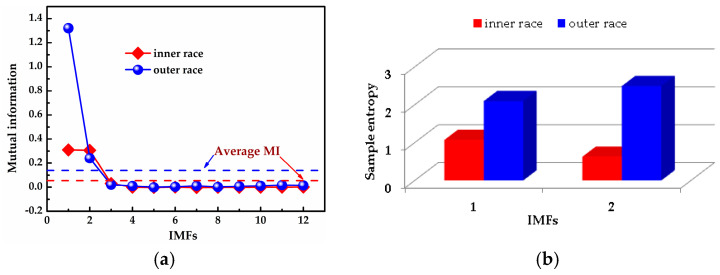
Calculation results of MI and SE: (**a**) calculation results of MI for all IMFs; (**b**) calculation results of SE for the “true” IMFs.

**Figure 18:**
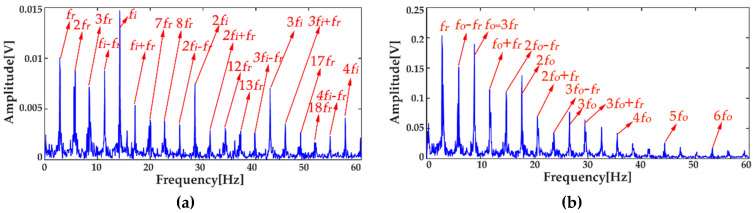
Envelope spectrums of the effective IMFs by MED-ICEEMDAN: (**a**) the result of the inner race; (**b**) the result of the outer race.

Figure 15 shows that the envelope spectra of the inner and outer races fault signals with MED de-noising were improved; compared with those without MED noise reduction, some fault features could be seen clearly. However, the multiple frequencies and side frequencies of the fault frequency were not shown completely, due to the noise pollution. The ICEEMDAN decompositions of the inner and outer races fault signals after MED noise reduction are shown in Figure 16a,b. The MI values between every IMF and the original signal are shown in Figure 17a. The average MI values of the inner and outer races were 0.0536 and 0.1387, which are indicated by red and blue dashed lines, respectively. The first two IMFs were selected as the “true” IMFs and their SE values are shown in Figure 17b.The effective IMFs of the inner and outer races fault signals were IMF2 and IMF1 according to the proposed MI-SE, and their envelope spectra are shown in Figure 18a,b, respectively. From Figure 18, it can be seen that the fault features of the inner and outer races, the multiple frequencies, and the side frequencies were almost all shown clearly by using the proposed scheme in this paper. The experimental results were in good agreement with the simulation results of Section 4, which further proves the feasibility of the method.

**Figure 19:**
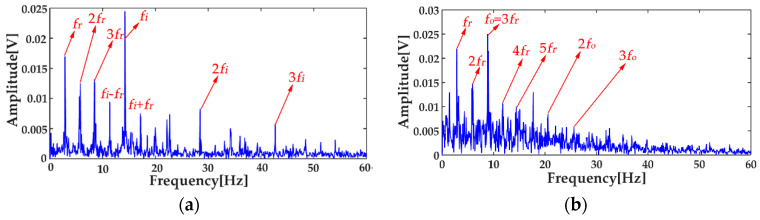
Results of the MED-adaptive multi-scale morphological analysis (AMMA): (**a**) the result of the inner race; (**b**) the result of the outer race.

In order to compare the proposed method, adaptive multi-scale morphological analysis (AMMA) was used to analyze the signals. The determination of the structural elements in AMMA is based on the local characteristics of the signal [24,25], and so it is adaptive and it effectively overcomes the randomness and subjectivity of the traditional mathematical morphology. Similarly, a hybrid fault features extraction method, MED-AMMA, is used to process the original signal of the bearing, and the results are shown in Figure 19. The fault frequencies of inner race and outer race can be seen; however, the results are not as ideal as that in the proposed method. The effectiveness of the proposed method is verified by the comparison between the two methods.

## 6. Conclusions

In this paper, a hybrid weak fault feature extraction method based on MED and ICEEMDAN is proposed, and a method to select a reliable and effective IMF based on MI-SE is also introduced. MED can increase the kurtosis value of the signal by more than three times, reducing the influence of the noise on ICEEMDAN. The MI-SE method provides a reliable criterion for selecting an effective IMF, and it can avoid the blindness of the traditional ICEEMDAN, which considers the first IMF as the effective IMF. The simulation and the experimental analysis that is presented in this paper demonstrate that the fault frequencies (including multiple frequencies and side frequencies) can be extracted successfully by the proposed method, compared with the ICEEMDAN or the MED.

Moreover, the scheme proposed in the paper provides a new idea for the weak fault features extraction of the slow speed and heavy load rolling bearings in a strong noise environment. In our next work, the proposed method will be used for the extraction of multiple faults and fault diagnosis.

## Figures and Tables

**Table 1:** The parameters of the rolling bearing.

Inside Diameter	Outside Diameter	Thickness	Ball Diameter	Pitch Diameter
17 mm	40 mm	12 mm	6.7 mm	28.5 mm
